# Continuous suprascapular nerve block compared with single-shot interscalene brachial plexus block for pain control after arthroscopic rotator cuff repair

**DOI:** 10.6061/clinics/2020/e2026

**Published:** 2020-11-02

**Authors:** Hoon Choi, Kyungmoon Roh, Mina Joo, Sang Hyun Hong

**Affiliations:** I Department of Anesthesiology and Pain Medicine, Seoul St. Mary’s Hospital, College of Medicine, The Catholic University of Korea, Korea; II Department of Anesthesiology and Pain Medicine, Eunpyeong St. Mary’s Hospital, College of Medicine, The Catholic University of Korea, Korea

**Keywords:** Arthroscopy, Postoperative Pain, Brachial Plexus Block, Anesthesia, Regional

## Abstract

**OBJECTIVES:**

We compared the analgesic efficacy of a continuous suprascapular nerve block (C-SSNB) and a single-shot interscalene brachial plexus block (S-ISNB) for postoperative pain management in patients undergoing arthroscopic rotator cuff repair.

**METHODS:**

A total of 118 patients undergoing arthroscopic rotator cuff repair were randomly allocated to the S-ISNB or C-SSNB groups. Postoperative pain was assessed using the visual analog scale (VAS) at 1, 2, 6, 12, and 24 h postoperatively. Supplemental analgesic use was recorded as total equianalgesic fentanyl consumption.

**RESULTS:**

The C-SSNB group showed significantly higher VAS scores at 0−1 h and 1−2 h after the surgery than the S-ISNB group (4.9±2.2 *versus* 2.3±2.2; *p*<0.0001 and 4.8±2.1 *versus* 2.4±2.3; *p*<0.0001, respectively). The C-SSNB group showed significantly lower VAS scores at 6−12 h after the surgery than the S-ISNB group (4.1±1.8 *versus.* 5.0±2.5; *p*=0.031). The C-SSNB group required significantly higher doses of total equianalgesic fentanyl in the post-anesthesia care unit than the S-ISNB group (53.66±44.95 *versus* 5.93±18.25; *p*<0.0001). Total equianalgesic fentanyl in the ward and total equianalgesic fentanyl throughout the hospital period were similar between the groups (145.99±152.60 *versus* 206.13±178.79; *p*=0.052 and 199.72±165.50 *versus* 212.15±180.09; *p*=0.697, respectively)

**CONCLUSION:**

C-SSNB was more effective than S-ISNB at 6−12 h after the surgery for postoperative analgesia after arthroscopic rotator cuff repair.

## INTRODUCTION

Shoulder surgery is related to severe postoperative pain (1). The use of arthroscopy is popular because it decreases pain, shortens hospital stay, and improves patient satisfaction. However, immediate postoperative pain remains to be a problem in more than 40% of patients (1,2). To date, the most effective method in controlling postoperative pain in shoulder surgery is regional nerve blocks, such as the interscalene brachial plexus block (ISNB) and the suprascapular nerve block (SSNB) (1,3).

ISNB is performed for shoulder surgery by injecting a local anesthetic or inserting a catheter near the brachial plexus for a continuous infusion (1,3). Single-shot ISNB (S-ISNB) is the most widely used technique because of its simplicity, but it is only effective for the first few hours. After approximately 6−8 h, S-ISNB results in severe rebound pain associated with decreased sleep quality and decreased patient satisfaction (4). Continuous ISNB (C-ISNB) is reportedly the most effective pain control technique for all types of shoulder surgery (1,3). However, maintaining the catheter around the brachial plexus can be technically difficult, as migration remains a problem (5,6). Moreover, adverse effects of ISNB, such as inadvertent epidural and spinal anesthesia, vertebral artery injection, paralysis of the vagus, recurrent laryngeal, and cervical sympathetic nerves, can be prolonged with C-ISNB (7). In addition, hemidiaphragmatic palsy, which can be a big problem in patients with respiratory disease, can be observed in almost all patients (8-10).

SSNB is an effective alternative method of regional nerve block in shoulder surgery (11,12). SSNB provides superior analgesia compared to placebo or local anesthetic infiltration and is considered noninferior compared to ISNB (13-16). The main advantage of SSNB is that it results in fewer adverse effects than ISNB, especially in hemidiaphragmatic palsy (13-16). Additionally, the catheter lies deep in the muscle layer and does not easily migrate, even when the patient’s neck moves (17,18).

In the present study, we compared the analgesic efficacy of continuous SSNB (C-SSNB) with S-ISNB for postoperative pain management within 24 h postoperatively in patients undergoing arthroscopic rotator cuff repair. Our primary hypothesis was that C-SSNB would be more effective than S-ISNB beyond 6 h after surgery.

## MATERIAL AND METHODS

The Institutional Review Board of Seoul St. Mary’s Hospital, College of Medicine, The Catholic University of Korea, approved this study (KC13OISI0623). All patients participating in this study provided written informed consent. Patients were enrolled in this study from December 2013 to August 2014 if they met all the following criteria: age 18-65 years; American Society of Anesthesiologists (ASA) status class I-II; and scheduled for arthroscopic rotator cuff repair with acromioplasty. Patients were excluded if they met any of the following criteria: ASA status >II; body mass index >35 kg/m^2^; or history of narcotic abuse, drug dependency, allergic reactions to local anesthetics, severe respiratory disease, proven coagulopathy, major neurologic deficits, or mental impairment.

This study was a prospective, balanced (1:1), randomized controlled parallel group trial. No changes were made to the design or protocol during the study. The patients were randomly allocated to one of two groups: S-ISNB and C-SSNB. Randomization was performed using a computer-generated randomization sequence with random block sizes of two and four, by a single nurse who was not involved in patient care. All surgical procedures were performed by the same surgeon. S-ISNB was conducted by the attending anesthesiologist, and C-SSNB was conducted by the surgeon who performed the surgery.

Standard ASA monitors were used throughout the surgery. General anesthesia was induced with intravenous propofol and remifentanil, which were also used for intraoperative maintenance of sedation as a continuous infusion using a target-controlled infusion device (Orchestra Base Primea, Fresenius Kabi, France) with a 40% oxygen-air mixture. Tracheal intubation was facilitated with 0.6 mg/kg of rocuronium. Long-duration opioids were avoided. A 0.3 mg of ramosetron was administered to patients 30 min before the end of the surgery for prophylaxis of postoperative nausea and vomiting.

All patients underwent arthroscopic rotator cuff repair in the lateral decubitus position. The undersurface of a type I acromion was smoothed. An acromioplasty was performed for type II and type III acromions along with the removal of subacromial spurs. The rotator cuff was repaired using suture anchors, and the single-row or double-row technique was selected according to tear size and torn cuff mobility. All portals made during the surgery were closed in a simple manner, and all patients were fitted with an abduction brace directly after the procedure.

S-ISNB was performed by the attending anesthesiologist via ultrasound (S-Nerve ultrasound, SonoSite, Bothell, WA, USA). At the end of the surgery and before emergence from anesthesia, the skin was prepared with chlorhexidine alcohol, and the nerve was located using a 38 mm, 6.0-13.0 MHz linear probe. Starting from the midline at the sixth and seventh cervical vertebra (C6, C7) level, the probe was moved laterally until the brachial plexus was detected between the anterior and middle scalene muscles. Then, the probe was moved following the route of the brachial plexus toward the nerve roots exiting the intervertebral foramen. After identifying the C5 and C6 nerve roots, the probe was moved back to the scalene muscles following the nerve roots, and 10 mL of 0.5% ropivacaine was injected between the C5 and C6 nerve trunks using an in-plane approach.

C-SSNB was performed by the surgeon via a nerve stimulator (TOF Watch, Organon, Swords Co., Dublin, Ireland) using a technique described by Checcucci et al. (12). At the end of the surgery and before emergence from anesthesia, an 18-gauge insulated Tuohy needle (Contiplex Tuohy set; B. Braun, Melsungen, Germany) connected to a nerve stimulator was inserted at a point 2 cm medial to the medial border of the acromion and 2 cm cephalad to the superior margin of the scapular spine. The needle was advanced toward the scapular notch while observing the supraspinatus and infraspinatus motor response (arm abduction and external rotation) elicited by the nerve stimulator. The current of the nerve stimulator was initially set to 2 mA, and the catheter was inserted at a point where the motor response was present at 0.5 mA but absent at 0.2 mA. After negative aspiration, 10 mL of 0.5% ropivacaine was injected through the catheter as a loading dose. All catheters were secured with cutaneous adhesive sutures and an occlusive dressing (Tegaderm; 3M Corp., St. Paul, MN, USA). An elastomeric pump (Homepump Infusion System; I-Flow Corp., Lake Forest, CA, USA) was connected to the catheter, and 0.25% ropivacaine was injected continuously at a rate of 2 mL/h for 24 h postoperatively.

Postoperative pain was additionally controlled with rescue analgesics. In the post-anesthesia care unit (PACU), patients with a visual analog scale (VAS) score ≥4 were initially given 50 µg of fentanyl and 30 mg of ketorolac intravenously. If more analgesia was needed, 50 µg of fentanyl was injected with a minimum duration of 10 min per injection. The patients were discharged from the PACU based on the modified Aldrete score (score ≥9). Patients in the ward with a VAS score ≥4 were given 25 mg tramadol intravenously with a minimum duration of 30 min per injection. If the pain did not subside after two repeated tramadol injections, 12.5 mg of pethidine was injected intravenously with a minimum duration of 30 min per injection.

The VAS score at movement was used to evaluate analgesic efficacy. The patients’ highest VAS score was recorded at 0−1 h, 1−2 h, 2−6 h, 6−12 h, and 12−24 h after arrival at the PACU (0 h). Preoperative VAS was obtained a day before the surgery. Preoperative demographic data and intraoperative findings were retrieved from electronic medical records. Postoperative consumption of analgesics was recorded as total equianalgesic fentanyl consumption: 25 mg of tramadol was considered equal to 50 µg of fentanyl, and 12.5 mg of pethidine was considered equal to 16.75 µg of fentanyl (19,20).

The primary endpoint of the present study was to compare the analgesic efficacy of C-SSNB with that of S-ISNB at 6−12 h after the surgery. The sample size was calculated based on a mean pain score of 5.2±2.9 at 6−12 h after ISNB for arthroscopic rotator cuff repair (21). We considered that a 30% decrease in the mean pain score was clinically significant (22,23). As 56 patients per group were required (alpha value of 0.05 and a power of 80%), we decided on a total of 61 patients, considering a 10% dropout rate. Comparisons of continuous values between the two groups were performed using Student’s t-test. The normality of the data distribution was assessed with the Shapiro-Wilk test. The results are presented as mean±standard deviation. Categorical variables, such as proportions, were compared between the groups using the chi-squared or Fisher’s exact test. A *p-*value of less than 0.05 was considered significant. All tests were performed using SPSS 21.0 software (IBM Corp., Armonk, NY, USA).

## RESULTS

**Figure 1:**
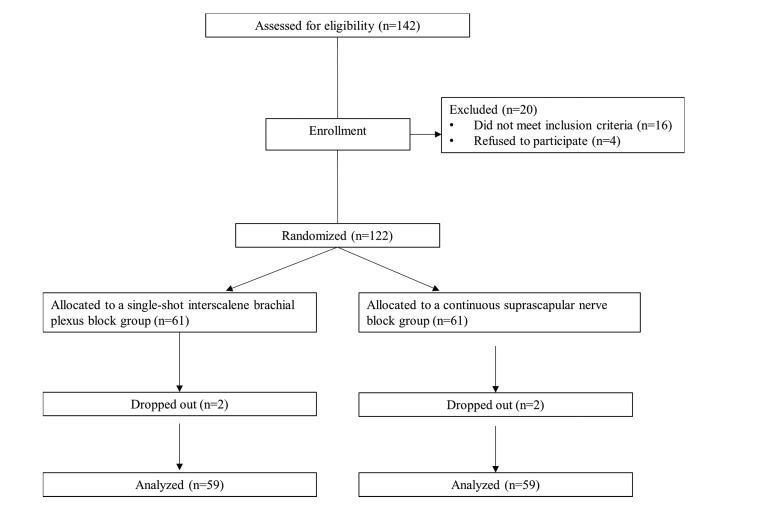
Consolidated Standards of Reporting Trials (CONSORT) guideline flow diagram.

A total of 122 patients were enrolled in the study. Two patients from each group dropped out after enrollment. Thus, 59 patients received S-ISNB, and 59 patients received C-SSNB (Figure 1). The demographic data, including age, height, weight, BMI, and sex ratio, were similar between the groups (Table 1). The profiles associated with the operation, such as preoperative VAS score, operation time, rotator cuff tear size, and anchor number, were similar between the groups (Table 2).

**Figure 2:**
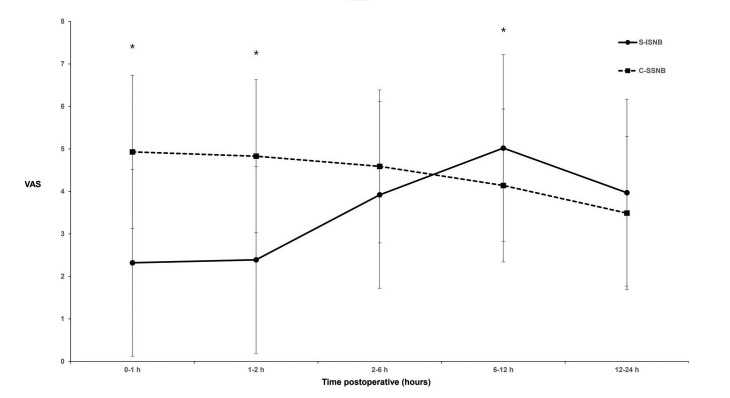
Graph shows changes in the visual analog scale (VAS) pain score after arthroscopic rotator cuff repair. Error bars indicate standard deviation. *indicates *p*<0.05 (significant difference).

The patients in the C-SSNB group showed significantly higher VAS scores at 0−1 h and 1−2 h after the surgery than the patients in the S-ISNB group (4.9±2.2 *versus* 2.3±2.2; *p*<0.0001 and 4.8±2.1 *versus* 2.4±2.3; *p*<0.0001, respectively; Figure 2). The mean difference in VAS scores at 0−1 h and 1−2 h was 2.6 (95% confidence interval [CI], 1.8 to 3.4) and 2.4 (95% CI, 0.4 to 1.6). The pain scores at 2−6 h after the surgery were similar between the groups (4.6±1.8 *versus* 3.9±2.7; *p*=0.109), with a mean difference of 0.7 (95% CI, -0.2 to 1.5). Patients in the C-SSNB group showed significantly lower VAS scores at 6−12 h after the surgery than the patients in the S-ISNB group (4.1±1.8 *versus* 5.0±2.5; *p*=0.031). The mean difference in VAS scores at 6−12 h was -0.9 (95% CI, -1.7 to -0.1). The pain scores at 12−24 h after the surgery were similar between the groups (3.5±1.7 *versus* 4.0±2.4; *p*=0.220), with a mean difference of -0.5 (95% CI, -1.2 to 0.3).

Patients in the C-SSNB group required significantly higher doses of total equianalgesic fentanyl in the PACU than patients in the S-ISNB group (53.66±44.95 *versus* 5.93±18.25; *p*<0.0001; Table 3). Total equianalgesic fentanyl in the ward and total equianalgesic fentanyl throughout the hospital period were similar between the groups (145.99±152.60 *versus* 206.13±178.79; *p*=0.052 and 199.72±165.50 *versus* 212.15±180.09; *p*=0.697, respectively)

## DISCUSSION

This study aimed to compare the analgesic efficacy of C-SSNB with that of S-ISNB for postoperative pain management within 24 h postoperatively in patients undergoing arthroscopic rotator cuff repair. The results suggested that C-SSNB was less effective than S-ISNB during the first 2 h after surgery. However, in the C-SSNB group, the pain score decreased gradually, but in the S-ISNB group, it increased gradually showing peak at 6−12 h after the surgery. C-SSNB was more effective than S-ISNB at 6−12 h after the surgery.

Postoperative analgesia for shoulder surgery is important for early recovery, rehabilitation, and patient satisfaction (1,3,15). Numerous methods have gained popularity, but all involve the dilemma of efficacy *versus* adverse effects (4). The two most popular methods are ISNB and SSNB (1,3,15). Many studies have confirmed the efficacy of these two methods compared to control, intravenous patient-controlled analgesia (IV-PCA), and local anesthetic infiltration (5-7,11,16,). Although many studies have compared the analgesic efficacy of SSNB and ISNB (2,17,21,27-39), this is the first study to compare C-SSNB and S-ISNB with rebound pain at 6-12 h after surgery as a primary endpoint.

In the current study, ISNB was performed as a comparator block via ultrasound, and 10 mL of 0.5% ropivacaine was injected once as a single bolus. One study reported that the success rates, as well as sensory, motor, and extent of the blockade, were significantly better in an ultrasound group than a nerve stimulator group (40). A continuous injection compared to a single bolus injection has generated conflicting results in terms of block success rate, efficacy, and additional side effects associated with catheters. Several studies have reported decrease in pain score and analgesic consumption with continuous injection (1,3,5,41). However, one study reported that placing a catheter for continuous ISNB is a time-consuming procedure with a low success rate (81%) (5). The dose used in the current study was the most widely accepted dose of S-ISNB for arthroscopic shoulder surgery (1). Our results revealed that S-ISNB provided effective analgesia during the first 6 h after the surgery, which was consistent with previous studies (4-6,24).

In contrast, SSNB was performed via a nerve stimulator, and a loading dose of 10 mL of 0.5% ropivacaine and a continuous dose of 0.25% ropivacaine at a rate of 2 mL per hour were injected through an implanted catheter. Many techniques have been studied to perform SSNB, but a nerve stimulator yields better results than blind ultrasound, fluoroscopy, and computed tomography-guided techniques (12,42-44). Direct arthroscopically guided SSNB has been introduced recently, but application of this method as preemptive analgesia is limited (35). The use of a catheter for SSNB has been reported in arthroscopic surgery (18, 45). Although the optimal dose needs to be verified in the future, a continuous dose of 2 mL per hour was sufficient to cover the small space in the scapular notch (2). As expected, C-SSNB resulted in a gradual decrease in the pain score over time and provided effective analgesia for at least 24 h after the surgery. These catheters lie deep in the muscle layer and do not easily migrate, even when the patient’s neck moves (17,18).

The main advantage of C-SSNB over S-ISNB was analgesia at 6−12 h after the surgery. The duration of analgesia from S-ISNB does not exceed 6-8 h postoperatively, and this results in extreme rebound pain resulting in poor sleep quality and decreased patient satisfaction (4). SSNB can be an alternative with a duration >6 h postoperatively (21,33). However, one study reported that the duration of SSNB is shorter than that of ISNB (2.53±2.26 h *versus* 7.23±6.83 h) (2). Considering these conflicting results, C-SSNB was selected in the current study. C-ISNB is limited by a low success rate (5), but in the case of C-SSNB, the catheter lies deep in the muscle layer and does not easily migrate (17,18). Considering the time course of pain control with C-SSNB and S-ISNB, a combination of the two methods needs evaluation in future research to develop an optimal option of consistent pain control after arthroscopic rotator cuff repair.

Although previous studies have reported that adding an axillary nerve block to SSNB improves shoulder analgesia (12,21,42,46-49), we did not apply the axillary nerve block. SSNB is known to be inferior compared to ISNB (33,35). This is because the suprascapular nerve originates from the upper trunk (C5 and C6 roots) of the brachial plexus, thus providing sensory fibers to 70% of the shoulder joint, but sensation in the remainder of the shoulder joint is provided by the axillary and lateral pectoral nerves (12). However, we wanted to focus on a simple and effective pain control method for patients undergoing arthroscopic rotator cuff repair that can be performed even in the surgical field.

This study had several limitations. First, the absence of a control group could lead to bias among the patients and investigators. We considered the S-ISNB group as an active comparator. A true placebo comparator would result in poor pain control, which would be unethical. Moreover, there could have been bias from the fact that none of the patients in the S-ISNB group had a catheter. Ideally, to secure the blindness of an investigator, we should have set up four separate groups as two factors were changed: single *versus* continuous, and interscalene brachial plexus *versus* suprascapular nerve. However, applying two catheters to all patients would have been difficult because of ethical reasons. Second, all blocks were done postoperatively. Preemptive analgesia plays an important role in postoperative analgesia. Additionally, the assessment of the blocks relied on a pain score, as sensory and motor testing was not possible during the immediate postoperative period. However, a preoperative catheter for SSNB can interfere with the surgery, and this issue needs cooperation from the surgeon. Third, the optimal dose for C-SSNB is lacking. As noted above, we found that a dose of 2 mL per hour was effective, but this requires verification and more research is needed to assess different doses and the quality of the block. Fourth, S-ISNB was done by the anesthesiologist, and C-SSNB was done by the surgeon. In addition, S-ISNB was done with ultrasound only, and C-SSNB was done with neurostimulation only. This could have increased bias, as mastery of the blocks may have been different between the two. However, we wanted to test whether C-SSNB can be easily and effectively performed in the surgical field immediately after completion of surgery. Fifth, we were not able to collect data on complications such as hemidiaphragm palsy.

## CONCLUSIONS

Postoperative pain control after arthroscopic rotator cuff repair was more effective with C-SSNB than S-ISNB at 6−12 h after the surgery.

## AUTHOR CONTRIBUTIONS

Choi H and Hong SH provided substantial contributions to the concept, design, investigation, drafting, and critical revision of the manuscript for important intellectual content. Roh K and Joo M provided substantial contributions to the design, investigation, and critical revision of the manuscript. All authors agreed to be accountable for all aspects of the work to ensure that questions related to the accuracy or integrity of any part of the study are appropriately investigated and resolved, and all authors participated sufficiently in the work to take public responsibility for appropriate portions of the content.

## Figures and Tables

**Table 1:** Comparison of demographic data.

Variables	C-SSNB (n=59)	S- ISNB (n=59)	*p* value
Age, year	55±12	57±8	0.277
Height, cm	161.4±8.9	161.6±8.3	0.890
Weight, kg	63.9±10.0	62.3±11.1	0.470
Body mass index, kg/m^2^	24.47±2.88	23.81±3.06	0.230
Male/female	25/34	31/28	0.269

C-SSNB, continuous suprascapular nerve block group; S-ISNB, single-shot interscalene brachial plexus block group.

Data are presented as mean±standard deviation or as number of patients.

**Table 2:** Comparison of profiles associated with the operation.

Variables	C-SSNB (n=59)	S-ISNB (n=59)	*p* value
Preoperation VAS	2.1±1.4	2.3±1.3	0.483
Operation time, min	134±23	136±35	0.731
Rotator cuff tear size, cm	1.25±0.65	1.33±0.78	0.521
Number of anchors	2±1	2±1	0.897

C-SSNB, continuous suprascapular nerve block group; S-ISNB, single-shot interscalene brachial plexus block group; VAS, visual analog scale.

Data are presented as mean±standard deviation.

**Table 3:** Supplement analgesia.

Variables	C-SSNB (n=59)	S-ISNB (n=59)	Mean difference (95% CI)	*p* value
PACU equianalgesic fentanyl dose, µg	53.66±44.95	5.93±18.25	47.73 (35.15, 60.30)	< 0.0001*
Ward equianalgesic fentanyl dose, µg	145.99±152.60	206.13±178.79	-60.14 (-120.75, 0.47)	0.052
Total equianalgesic fentanyl dose, µg	199.72±165.50	212.15±180.09	-12.43 (-75.50, 50.64)	0.697

C-SSNB, continuous suprascapular nerve block group; S-ISNB, single-shot interscalene brachial plexus block group; PACU, post-anesthetic care unit.

Data are presented as mean±standard deviation.

* *p*<0.05 is statistically significant.
